# LCF and HCF of Short Carbon Fibers Reinforced AE42 Mg Alloy

**DOI:** 10.3390/ma16103686

**Published:** 2023-05-12

**Authors:** Naser A. Alsaleh, Sabbah Ataya, Fahamsyah H. Latief, Mohamed M. Z. Ahmed, Ahmed Ataya, Akrum Abdul-Latif

**Affiliations:** 1 Department of Mechanical Engineering, College of Engineering, Imam Mohammad Ibn Saud Islamic University, Riyadh 11432, Saudi Arabia; naalsaleh@imamu.edu.sa (N.A.A.); smataya@imamu.edu.sa (S.A.); 2 Department of Metallurgical and Materials Engineering, Faculty of Petroleum and Mining Engineering, Suez University, Suez 43512, Egypt; 3 Department of Mechanical Engineering, Faculty of Engineering and Science, Universitas Nasional, Jakarta 12520, Indonesia; fhlatief@civitas.unas.ac.id; 4 Department of Mechanical Engineering, College of Engineering at Al Kharj, Prince Sattam bin Abdulaziz University, Al Kharj 11942, Saudi Arabia; 5 Department of Materials Science & Engineering, Texas A&M University, College Station, TX 77843, USA; a.ataya@tamu.edu; 6 Université Paris 8, IUT de Tremblay, 93290 Tremblay-en-France, France; akrum.abdul-latif@univ-paris8.fr

**Keywords:** LCF, HCF, tensile, fracture, AE42, carbon fiber, composite

## Abstract

Lightweight magnesium alloys and magnesium matrix composites have recently become more widespread for high-efficiency applications, including automobile, aerospace, defense, and electronic industries. Cast magnesium and magnesium matrix composites are applied in many highly moving and rotating parts, these parts can suffer from fatigue loading and are consequently subjected to fatigue failure. Reversed tensile-compression low-cycle fatigue (LCF) and high-cycle fatigue (HCF) of short fibers reinforced and unreinforced AE42 have been studied at temperatures of 20 °C, 150 °C, and 250 °C. To select suitable fatigue testing conditions, tensile tests have been carried out on AE42 and the composite material AE42-C at temperatures of up to 300 °C. The Wohler curves *σ_a_ (N_F_)* have shown that the fatigue strength of the reinforced AE42-C in the HCF range was double that of unreinforced AE42. In the LCF range at certain strain amplitudes, the fatigue life of the composite materials is much less than that of the matrix alloys, this is due to the low ductility of this composite material. Furthermore, a slight temperature influence up to 150 °C has been established on the fatigue behavior of the AE42-C. The fatigue life curves Δ*ε_total_ (N_F_)* were described using the Basquin and Manson–Coffin approaches. Fracture surface investigations showed a mixed mode of serration fatigue pattern on the matrix and carbon fibers fracturing and debonding from the matrix alloy.

## 1. Introduction

The demands for weight reduction in product engineering development, particularly in the transportation and automotive industries, have increased over time, causing intensive efforts to be made relentlessly. This weight reduction greatly affects emission reduction [1], which has a positive impact on environmental factors and, moreover, will also bring benefits from an economic perspective [2]. Reflecting on recent trends, the utilization of magnesium alloys has been used in automotive components [3], and it has even been reported that several types of magnesium alloys have begun to be used in the manufacturing industry for applications in electric vehicle chassis [4] and body structures [5]; this has attracted the attention of scientists worldwide to develop magnesium-based alloys and their composites for many applications. The choice of magnesium as a potential candidate for engineering applications is due to its good castability and machinability, high specific strength, and, more uniquely, well-controlled weldability [5,6].

Despite their advantages, magnesium alloys also have poor corrosion resistance and low performance at elevated temperatures [7,8]; the previous results reported that magnesium alloys have low mechanical strength and relatively low wear resistance, which ultimately restricts their use in anti-friction and structural applications [9]. In fact, one of the major issues in recent decades has been the low creep resistance of magnesium alloys, which limits their range of applications. Mo et al. [10] stated that the content of alloying elements in magnesium alloys influenced the low creep resistance. It has also been established that the improvement in mechanical properties and stability in magnesium alloys for elevated temperature applications can be achieved by adding rare earth elements [11,12].

Not only that, there are other methods that are also usually undertaken to enhance the mechanical properties of magnesium alloys, namely by adding reinforcing materials to magnesium alloys to make magnesium alloy-based composites in order to upgrade their mechanical properties as required. Referring to several studies that have been conducted previously, incorporating reinforcement materials into magnesium alloys in the form of SiO_2_ nanoparticles [13], carbon nanotubes/CNTs [14], and short fibers of Al_2_O_3_ [15] or carbon [16] can considerably improve their mechanical strength [15,17] and creep resistance [18]. Indeed, short carbon fiber reinforcement has the advantages of increasing stiffness, elastic modulus, and creep resistance while maintaining a light weight, opening up opportunities to expand its use in different engineering applications [19]. In addition, Xu et al. [20] noted that as the content of SCFs increases, mechanical properties decrease, the grain size of the composite increases, and the degree of aggregation increases. In the early stage of reinforcement, the mechanical properties increased, followed by the decrease in mechanical properties with the increase in the content of SCFs from the point of view of mechanical performance. Moreover, Ataya et al. [21] evaluated the strength and wear resistance of AE42 magnesium alloy reinforced with short carbon fibers; the results revealed that the yield stress and compressive strength increased significantly at different temperatures. Even the wear resistance of the magnesium-based composite was better when compared with magnesium alloy.

For further consideration, in many technical applications (e.g., engine components and load-bearing structures) [18], the manufactured products must have good resistance to a high number of dynamic load cycles without failure. This is due to the placement of engine components surrounded by detrimental environments such as elevated temperatures which can negatively impact the fatigue behavior of magnesium alloys. This phenomenon causes the fatigue behavior of magnesium alloys to be reevaluated further to yield products fit enough for use in such conditions. Teschke et al. [22] observed the high-temperature compression–compression fatigue behavior of conventional AE42 magnesium alloy containing rare earth elements and DieMag422 magnesium alloy without rare earth elements. Interestingly, in the compression–compression fatigue test, as the temperature increased, there was a decrease in the fatigue strength of both alloys; it was also found that the increase in temperature caused the compression fatigue strength of the DieMag422 alloy to be higher than that of the AE42 alloy. Meanwhile, Shih et al. [23] carried out rotational bending fatigue experiments on extruded AZ61A magnesium alloy. From the results of their study, it was found that fatigue strength was predicted at 10^7^ cycles, with probabilities varying between 10 and 90%. As for crack initiation, it starts from inclusions present on the surface of the specimen or in the area near the surface, and the microstructure of the Mg alloy strongly influences the initial crack growth behavior. More recently, Ghorbanpour et al. [24] conducted a study on the influence of ambient temperature and specimen pre-straining on the high-cycle fatigue behavior of WE43-T5 magnesium alloy. Initially, this WE43-T5 alloy showed higher fatigue strength compared to most other magnesium alloys; however, it decreased with increasing the temperature from room temperature to 100 °C. Briefly, it is necessary to note specifically that structural parts and vehicle components are subjected to repeated loading under operating conditions; hence, the materials used should have excellent fatigue behavior in order to provide satisfaction in their use [25].

Additionally, there is a different type of Mg-Al alloy called AZ31B, which has been evaluated by fatigue testing at room temperature, 100 °C, and 200 °C with varying strain amplitudes. The results showed that increasing the temperature results in an increase in the plastic strain portion of the total strain, resulting in an increase in loop width. However, there is no inflection point in the cycle half-life at 200 °C at all strain amplitudes [26]. In addition, the MRI153 alloy developed by Volkswagen, consisting of Mg-Al-Ca-Re, has shown good performance in that it can operate for a long time under environmental conditions of 150 °C and 50–80 MPa without changing its casting conditions for the production of automotive driving parts. More surprisingly, MRI153 alloy performs better than AE42 and AZ91D under the same conditions and is used for automobile oil chassis parts and gearbox housings [27]. Kim et al. [28] have studied the effect of artificial cooling on the high-cycle fatigue characteristics of AZ91 alloy subjected to extrusion. The results obtained demonstrate that the artificially cooled AZ91 has better fatigue resistance than the one without artificial cooling. This is evident from the significantly smaller fatigue damage per cycle of the artificially cooled sample when compared to the one without artificial cooling.

In this study, the focus is on the fatigue of AE42 magnesium alloy reinforced by short carbon fibers due to the lack of research in this area. Alloy AE42 is a type of commercial magnesium alloy consisting of Mg-Al with added rare earth elements. The selection of AE42 magnesium alloy is motivated by its high creep resistance up to a temperature of 150 °C, this is attributed to its content, including 4 wt % Al and 2.5 wt % rare earth elements, thereby providing a strong reason for this AE42 alloy to be used for automotive engine components [29]. However, Wu et al. have investigated AE42 alloys treated with additional Ca where the yield stress increased at both room and elevated temperatures. Even the tensile strength of the AE42 alloy at elevated temperatures increases significantly with the addition of Ca [7]. Wittke et al. [30] have carried out studies comparing the fatigue strength between AE42 and Mg-4Al-2Ba-2Ca alloys at room temperature; the results showed that the fatigue strength of AE42 was about double that of Mg-4Al-2Ba-2Ca. With regard to testing under constant amplitude conditions, the difference in cyclic deformation behavior of the two types of alloys was observed, affecting the cyclic creep resistance and service life in the tensile loading interval at room temperature. Dieringa et al. [31] stated that the creep strength of AE42 is lower than that of DieMag422 at temperatures between 175 °C and 240 °C and at stresses between 60 MPa and 120 MPa. These facts further solidify AE42 as a potential candidate material for the manufacturing of automotive components. This triggers efforts to improve the fatigue properties of AE42 to be of interest in this study by adding short carbon fibers to produce magnesium composites with better properties to meet the requirements and criteria needed in the design of structural components and the prediction of their service lives.

Therefore, the present study was aimed at investigating the fatigue behavior of short carbon fibers reinforced with AE42 magnesium alloy in both LCF and HCF ranges at temperatures of up to 250 °C. Fatigue testing was carried out in reversed (tensile or compression) loading mode. To define the fatigue testing loads or strains, tensile tests were conducted on the unreinforced AE42 and the composite material AE42-C at temperatures up to 300 °C. In terms of the fracture surface, it has been reported that the morphology of the AE42 fracture surface looks rougher when compared to DieMag422 in the HCF regime. This is due to the high ductility of AE42, which makes it more difficult to recognize the fatigue crack propagation and final rupture areas [32]. The fracture surface of an alloy specimen after fatigue testing can be analyzed by the entire fracture surface method, whose function is to reduce the entire surface area associated with geometric discontinuities or missing points so as to achieve dimensional uniformity in all specimens [33,34]. Moreover, the fracture surfaces were observed using scanning electron microscopy to examine the fracture mode.

## 2. Materials and Methods

The material under investigation is the unreinforced AE42 and the reinforced material (AE42-C), which reinforced a high volume fraction of short carbon fibers. The carbon fibers (*L_f_* ~ 100 µm, and *d_f_* ~ 7 µm, volume faction *V_f_* = 0.23) are quasi-isotropically distributed in the reinforced plane. The chemical composition of the alloy AE42 is listed in Table 1.

Specimens were made from the composite material in such a way that the force was applied parallel to the reinforced plane during the test. Further information on the examined material is reported in [1,2]. Fatigue tests were carried out at temperatures of 20 °C, 150 °C, and 250 °C on short fiber reinforced and non-reinforced magnesium alloy AE42. Scanning electron microscopic (SEM) investigations were carried out on the fractured specimens using SEM Type LEO 1450 VP (Carl Zeiss, Jena, Germany) up to 30 kV, equipped with the EDS analyzer Type Oxford (Oxford Instuments, Oxford, UK).

**Figure 1:**
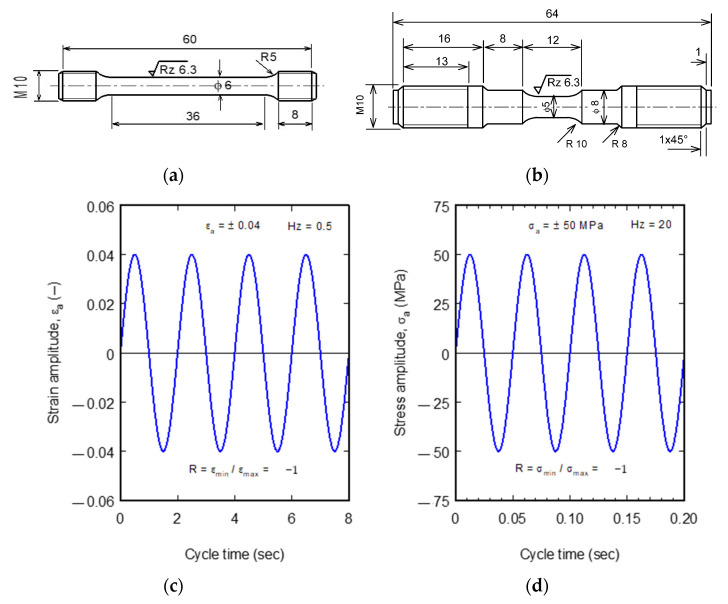
Specimen for tensile test and alternating tensile-compression fatigue test cycles. (**a**) Tensile test specimen according to ASTM E8; (**b**) Fatigue test specimen according to ASTM E466; (**c**) Example of strain-controlled cycle; (**d**) Example of stress-controlled cycle.

Figure 1 shows the tensile and alternating fatigue (*R* = −1) test specimens. Tensile specimens were machined in accordance with the small size specimen per the ASTM E8 standard [35] (Figure 1a), and fatigue specimens were fabricated in accordance with ASTM E466 [36,37] (Figure 1b).

Tensile and fatigue tests were conducted on the universal servohydraulic tensile testing machine Type MTS 810 (MTS Systems Corporation, Eden Prairie, MN, USA) with a maximum load of 100 kN and a maximum speed of 100 mm/s; the machine is equipped with a furnace with a maximum temperature of ≈800 °C. Tensile tests have been carried out at a strain rate of 0.001 s^−1^ (crosshead speed = 0.03 mm/s). The room temperature extension is measured using an extensometer of 0.5 µm accuracy with a maximum distance of 10 mm, while in the high-temperature tests, an extensometer with inductive rods and accuracy of 1 µm and a maximum distance of 40 mm was used. These extensometers have been used in the strain-controlled LCF range. Alternating strain-controlled fatigue tests were performed in the LCF range (*N* < 10^4^ cycles) at a test frequency of 0.5 Hz. Figure 1c shows the tests performed in the HCF range (*N* > 10^4^ cycles). At the same time, stress-controlled tests were carried out at frequencies of up to 50 Hz and load cycles of up to 2 × 10^7^. The frequency was increased with decreasing applied stress to shorten the time needed to conduct HCF tests. To reach 10^7^ cycles, it takes about 47 h at the maximum frequency (50 Hz), while at high stresses or strain-controlled tests, the frequency has been decreased to the lowest speed (0.5 Hz) to avoid damaging the extensometers. All tests’ alternating strain and stress ratio (minimum stress/maximum stress) was *R*= −1. Figure 1c shows an example of the strain-controlled test carried out at a strain amplitude of *ε_a_* = 0.04 and frequency of 0.5 Hz. An example of a stress-controlled test carried out at a stress amplitude of *σ_a_* = 50 MPa and frequency of 20 Hz is shown in Figure 1d. Fatigue tests were conducted until either the fracture of the specimen or they were terminated upon reaching the fatigue limit at 10^7^ cycles. The number of specimens (experiments) for each condition carried out to build the fatigue curve ranged between 13 and 21, with one test specimen at each selected stress or strain; however, two specimens were also used at some point, which showed a discrepancy in the results.

## 3. Results and Discussions

### 3.1. Microstructure

**Figure 2:**
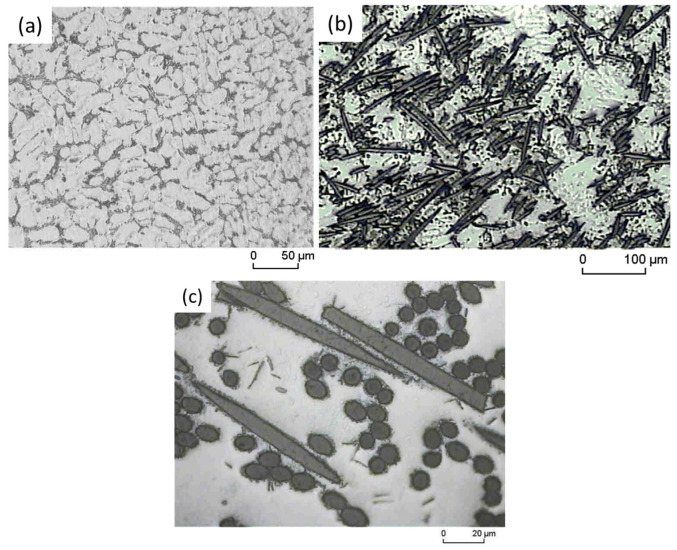
Optical microstructure of (**a**) the unreinforced AE42, (**b**) distribution of the fibers in the composite material AE42-C, and (**c**) closer image of the fibers.

Figure 2 shows the microstructure of the as-cast AE42 alloy and its composites. In general, the AE42 alloy consists mostly of an irregularly arranged cast α-Mg phase and some fine eutectic lamellas are randomly scattered at the grain boundaries, as seen in Figure 2a. In addition, it is common to observe the presence of another intermetallic phase of aluminum with rare earth elements (RE) recognized as Al_11_RE_3_, which is formed due to the addition of rare earth elements and is mainly located at the grain boundaries. The microstructural appearance as observed in this present study is in line with that previously confirmed by Wu et al. [38]. A similar intermetallic compound, Al_11_RE_3_, has also been detected by Xue et al. using XRD analysis of as-cast AE42 [39]. In the case of the AE42 composite, the purposely added short carbon fibers are almost evenly quasi-isotropically distributed in the reinforced plane of the AE42 alloy acting as the matrix (Figure 2b). In the high magnification image (Figure 2c), the short carbon fibers can be seen either cut longitudinally or through its fiber cross-section. The embedded fibers in the matrix show good compatibility with the matrix, indicating good wettability of the fibers by melting during the squeeze casting process, ultimately indicating the adequate formation of the composites AE42-C (Figure 2c).

### 3.2. Tensile Test Results

**Figure 3:**
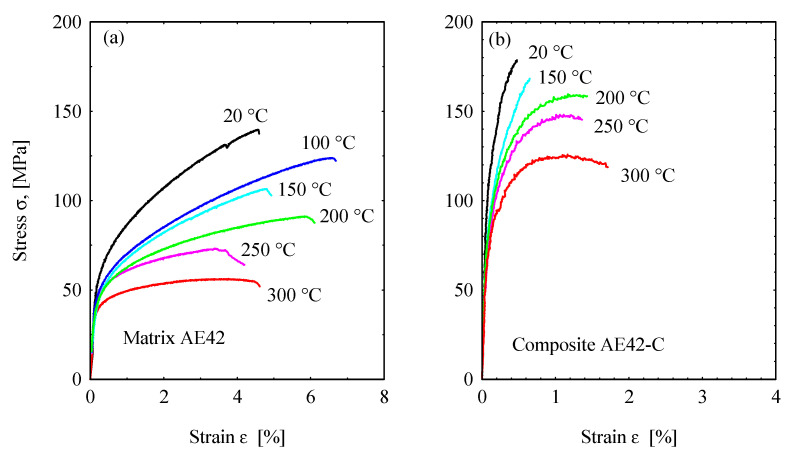
Tensile stress–strain curves of (**a**) the unreinforced AE42, and (**b**) the composite material AE42-C at different temperatures.

The stress–strain curves for AE42 and AE42-C at various temperatures are shown in Figure 3. Initially, the flow stress increases very abruptly at the beginning of the deformation process in both the unreinforced AE42 (Figure 3a) and the reinforced AE42-C (Figure 3b). This increase can be attributed to the work-hardening mechanism in the unreinforced AE42 induced by the accumulation of dislocations and kinks, in addition to the strengthening of the composite by the reinforcing carbon fibers. The maximum applicable stresses achieved at 20 °C in the AE42 and the composite are about 147 MPa and 180 MPa, respectively. Moreover, the stresses decreased with periodic increases in temperature applied during the tensile tests.

With regard to the increase in stress, it can be generally attributed to the work-hardening mechanism promoted by the accumulation of dislocations and their kinks [40]. Even more interestingly, in this study, work hardening is more pronounced at low temperatures [41]. Whereas the subsequent decrease in stress can be associated with the softening process caused by the rearrangement of dislocations and the gradual demolition of dislocations [42]. From the curves in Figure 3, it can also be understood that there is a typical stress behavior by combining the two opposing interactions of work hardening and dynamic softening, where temperature dependence is one of the factors that needs to be seriously accounted for. The incorporation of short carbon fibers has successfully improved the tensile strength of AE42 composites; however, it has caused the composites to become more brittle and fracture prematurely, as the strain values or ductility are relatively lower than that of the AE42 alloy (Figure 3b). Hence, the presence of elastic short carbon fibers in the composite restricts plastic deformation at different temperatures.

The tensile tested unreinforced AE42 was in the as-cast condition. The shortening in the tensile curves at high temperatures (150–300 °C) could be due to softening occurring in the eutectic structure at the grain boundaries; one of the purposes of conducting tensile tests is to determine the stress and strain for conducting the fatigue tests, this purpose can be fulfilled by the tensile curves up to low strain values. The maximum applied strain in the strain-controlled tests was *ε_a_* = 0.04 at 250 °C.

**Figure 4:**
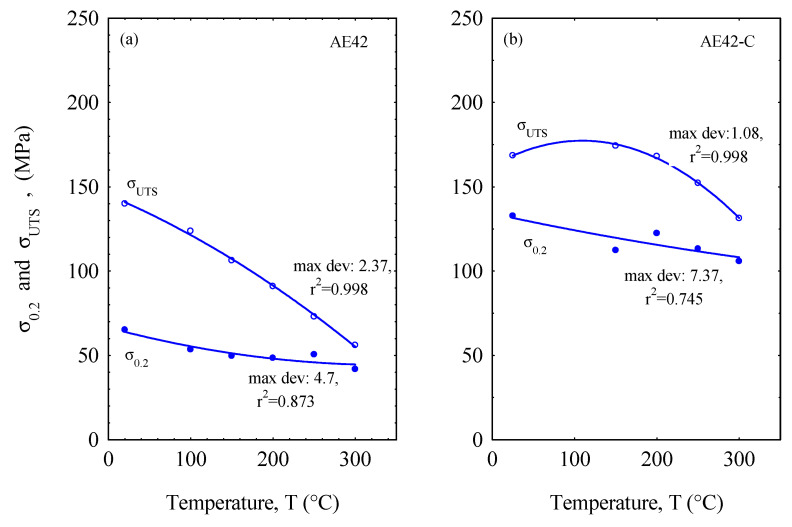
Tensile yield stress (σ_0.2_) and ultimate tensile strength (σ_UTS_) of (**a**) unreinforced AE42, and (**b**) AE42-C against the test temperatures.

The tensile yield stress and ultimate tensile strength values of the AE42 alloy and its composite are shown in Figure 4. The results in Figure 4 have been fitted using a second-order function and the maximum deviation standard error is presented on the curves. These results show a clear difference between the two materials, with the AE42-C composite showing higher results than the unreinforced AE42 as a function of increasing temperature. However, upon closer inspection, there is a drastic decrease in the ultimate tensile stress of AE42 as the temperature increases, while the decrease in the tensile yield stress of both materials has a similar trend, although the AE42-C composite has a better value. This is mainly due to the incorporation of short carbon fibers into the AE42 alloy, which causes the AE42 alloy—which acts as the matrix—to become brittle, resulting in a sharp increase in its ultimate tensile strength compared to the unreinforced AE42. The distribution and well-bonded nature of the short carbon fibers in the matrix were found to have a positive influence on the mechanical properties of the unreinforced AE42 [5,6]. Previous research has shown that carbon fibers can act as heterogeneous nucleation sites during the solidification of the magnesium alloy matrix. In the case of short carbon fibers distributed at grain boundaries, they can be a hindrance to grain growth, which also contributes to the improvement in mechanical properties [7,8]. In addition, the reaction that occurs at the interface between the matrix and the short carbon fiber forms brittle carbides, resulting in a strong interfacial bond (Figure 2c). Because of their brittleness and ability to transfer the subjected load, the carbides at the interface affect the mechanical properties of the composite [43,44,45,46,47].

**Figure 5:**
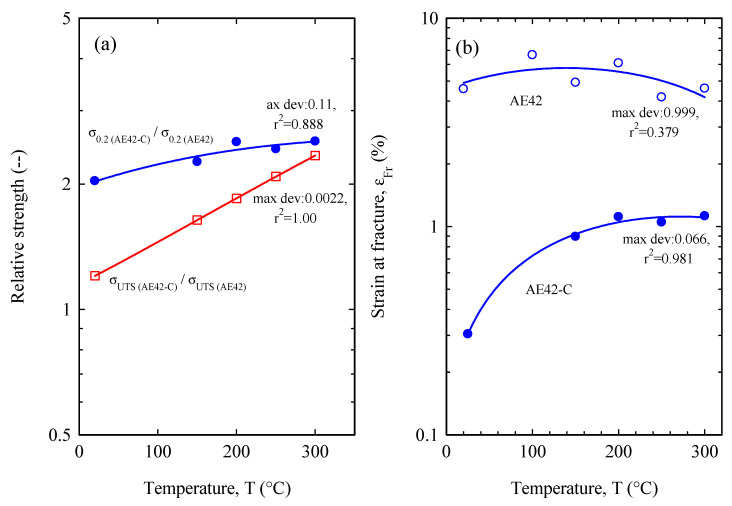
Tensile results of (**a**) relative strength and (**b**) strain at fracture of the unreinforced AE42 and AE42-C at different temperatures.

Figure 5 illustrates the relative yield stress (σ_0.2 (comp)_/σ_0.2 (matrix)_) and the relative ultimate tensile strength (σ_UTS (comp)_/σ_UTS (matrix)_) and fracture strain of the unreinforced AE42 and AE42-C. The results in Figure 5 have been fitted using a second-order function and the maximum deviation standard error is presented on the curves. Understandably, the composite’s relative strength versus its matrix illustrates an increase in both the tensile ultimate strength and the tensile yield stress (Figure 5a). Interestingly, the magnitude of the relative increase for the yield stress is much greater than that of the ultimate tensile strength under increasing temperature conditions. Because of the early fracture of the composite (Figure 3b) and the significant decrease in the ultimate tensile strength of the AE42, the relative improvement in the ultimate tensile strength seems relatively low at low temperatures. However, the relative ultimate tensile strength improves drastically with temperature due to the higher temperature resistance of the composites compared to the matrix alloy. The relative yield stress starts with values higher than two, which means that the material yield strength is clearly increased with reinforced carbon fibers. Moreover, the improvement in the relative tensile yield stress is more gently triggered by the factor of the very low tensile yield stress of the matrix, and thus the relative yield stress rises slightly with increasing temperature. Turning to the strain at fracture (Figure 5b), the AE42 alloy matrix is seen to be superior to its composite due to the effect that the addition of short carbon fibers induces, as mentioned earlier [48].

### 3.3. Wohler Curves of the Tested Materials

**Figure 6:**
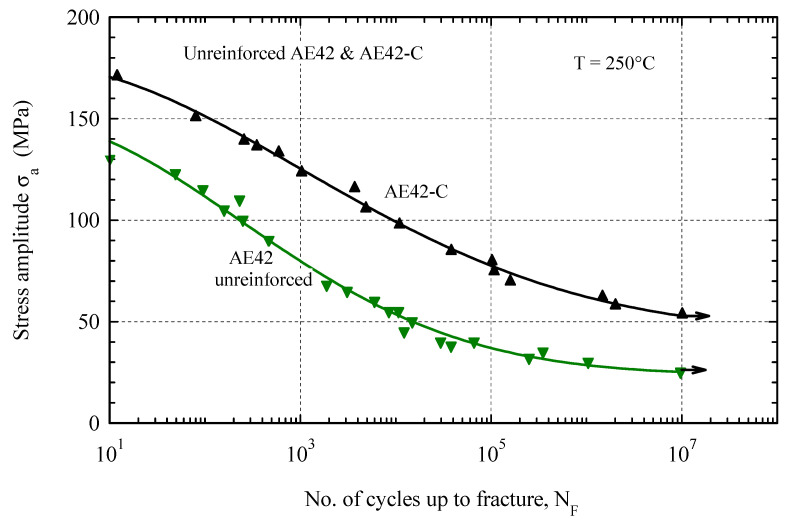
Wohler curves of the unreinforced AE42 and AE42-C at 250 °C.

Figure 6 shows the Wohler curves of the composite materials AE42-C and its matrix alloy at 250 °C. The composite AE42-C showed a higher fatigue resistance than the unreinforced alloy AE42. It is clear from Figure 6 that the fatigue strength of the AE42 composite is twice that of the AE42 alloy at N = 10^7^ cycles, where the fatigue strength is about 25 MPa for the AE42 alloy and 52 MPa for AE42 composite at 250 °C, respectively. This indicates that temperature is largely responsible for the decrease in fatigue strength in both materials. The arrow in Figure 6 at the number of cycles equal to 10^7^ indicates that these specimens are not fractured, but that the test has been terminated at this running life. The cause of the decrease in fatigue strength is due to the softening effect that occurs at 250 °C in both materials [49].

**Figure 7:**
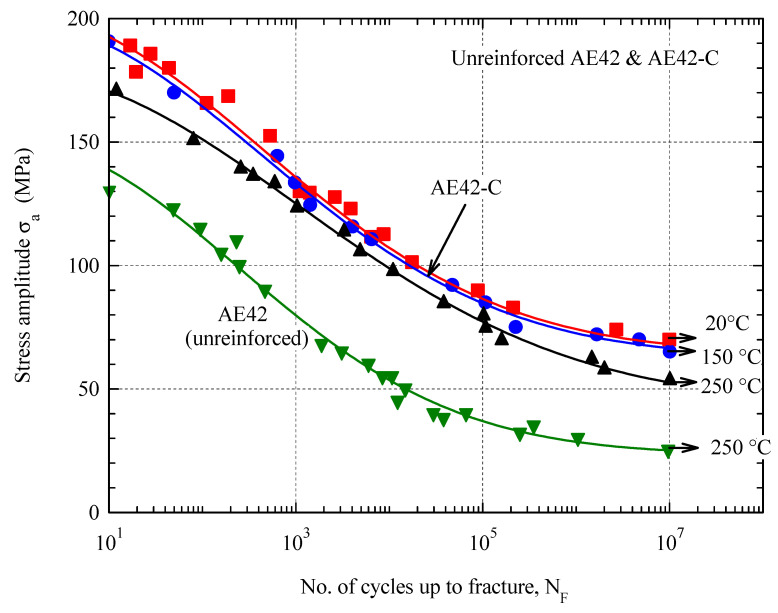
Wohler curves of AE42-C at 20 °C, 150 °C, and 250 °C compared with the unreinforced AE42 at 250 °C.

Increasing the temperature from 20 °C to 150 °C showed no significant effect on the fatigue behavior of the AE42-C composite, so the results at both temperatures could be described with a curve (Figure 7), although the results at room temperature were more scattered than at 150 °C. As the temperature increases to 250 °C, the fatigue strength decreases only slightly. This trend confirms the thermal stability of the AE42-C composite and its potential applicability at higher temperatures. The thermal stability is, on the one hand, based on the higher strength reinforcement material and, on the other hand, on the thermal stability of the matrix microstructure of unreinforced AE42 due to the stable intermetallic phase Al_11_RE_3_ (*T_S_* ≈ 1200 °C) [50]. Another factor causing the reduction in fatigue strength with increasing temperature may be the reduction in elastic modulus at higher temperatures [51].

In Figure 7, fatigue (S-N) curves have been fitted using an arbitrary exponential equation as described in Equation (1):

(1) $$\sigma_{a}=a\frac{\exp\left(-\log N\right)}{b^{2}}+c$$

Table 2 contains the fitting parameters at different temperatures. Fatigue strength has been determined from the fitting curve as the stress at maximum attained life ($N=10^{7}$ cycles). Fatigue strength is also listed in Table 2. However, reinforced and unreinforced AE42 alloy showed lower fatigue strength compared with some aluminum alloys. Murashkin et al. [52] reported that the fatigue strength of coarse-grained AA6061 at room temperature at 10^7^ cycles was 100 MPa, whereas the maximum achieved fatigue strength of the composite AE42-C was 68 MPa.

### 3.4. SEM Investigations

**Figure 8:**
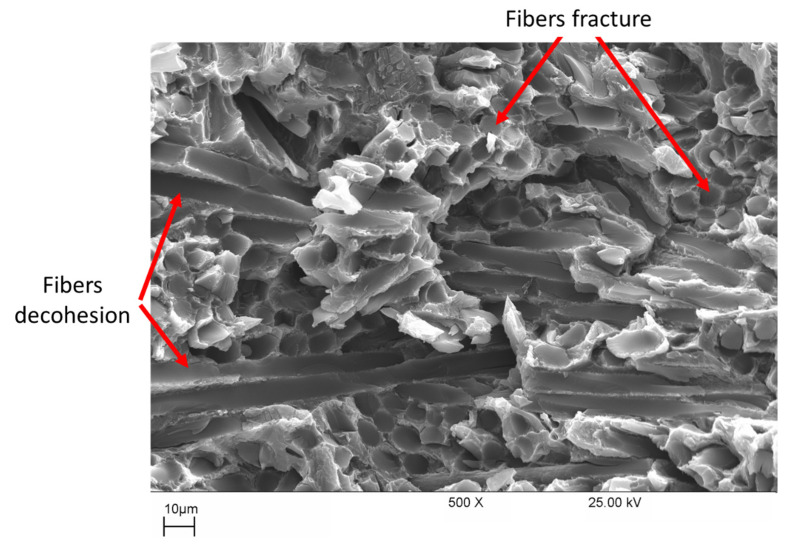
SEM micrograph of a fractured tensile specimen of the composite AE42-C at room temperature.

Figure 8 shows the SEM image of the fracture surface of a tensile specimen tested at room temperature. The fracture features can be summarized in the following: (i) fibers aligned with or tilted with the loading direction are fractured; (ii) fibers aligned perpendicular to the loading direction are subjected to decohesion from the unreinforced alloy, and the matrix material filling the spaces between the fibers is fractured after little deformation. This behavior, besides the high-volume fraction of fibers in the composites, explains the early fracture of composite tensile specimens after limited strain values, as shown in the tensile stress–strain curves presented in Figure 3b.

**Figure 9:**
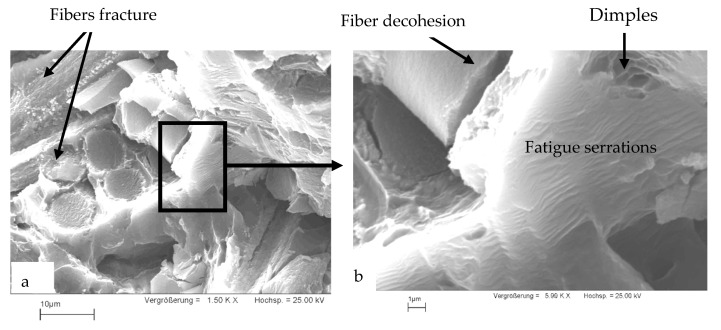
SEM micrograph of composite specimen fractured AE42-C at *σ_a_* ± 98.5 MPa, N = 4560 cycles, Hz = 0.5, and at 250 °C.

Figure 9 includes a SEM image of a fatigue specimen fractured at a stress amplitude of *σ_a_* ± 98.5 MPa after a fatigue life of N = 4560 cycles at a temperature of 250 °C. Fibers show a behavior similar to that shown in the specimen fractured under tensile load (Figure 8). Fibers parallel to the loading direction (Figure 9a) break in the transverse direction. Likewise, breaks along the fibers that are oriented perpendicular to the direction of loading can be detected. In addition, fiber detachment from the matrix could be observed on the fracture surfaces of the fatigue specimens. The matrix bridges that enclose the fibers behave like metallic materials under vibrational stress. The different stages of fatigue crack growth [53] have been observed in the metallic bridges in the crack initiation phase. Figure 9b shows pronounced vibrating stripes, which can be traced back to the fatigue loading. In the end, the metallic bridges break violently, which is characterized by a dimpled structure.

### 3.5. Fatigue Life Estimation

There are models describing the strain-controlled low-cycle fatigue life curves *ε (N_F_).* Some of these models propose the strain amplitude consists of an elastic part *ε_el_* and a plastic part *ε_pl_*. The double-logarithmic representation of the dependency *ε (N_F_)* can be described with a combination of two approaches. The elastic part *ε_el_* is described using Basquin’s Equation (2) [54], while the plastic part *ε_pl_* is expressed using the Manson–Coffin relationship [55,56] (Equation (3)).

(2) $$∆\varepsilon_{el}=AN_{F}^{-n}$$

(3) $$∆\varepsilon_{pl}=B\;N_{F}^{-m}$$

The summation of both parts ($∆\varepsilon_{el}\;$and $∆\varepsilon_{pl}$) results in total strain $\left(∆\varepsilon_{t}\right)\;$via the following relation:

(4) $$∆\varepsilon_{t}=A\;\;N_{F}^{-n}+B\;\;N_{F}^{-m}$$

According to general theoretical approaches [55,56,57], values *n =* 0.10 and *m =* 0.5 can be assumed for *n* and *m*; however, the use of such constants can lead to larger deviations from the actual material behavior. The coefficients *A* and *B* can be determined by the following relationship.

(5) $$A=\xi \;\frac{\sigma_{UTS}}{E}$$

where E and $\sigma_{UTS}$ are temperature-dependent elastic modulus and ultimate tensile strength, respectively. The parameter ξ is often set to 3.5 [55,58]. The parameter *B* can be calculated from the reduction in the area at the break determined in the tensile test [59] as follows,

(6) $$B=-In\;\left(1-Z\right)$$

**Figure 10:**
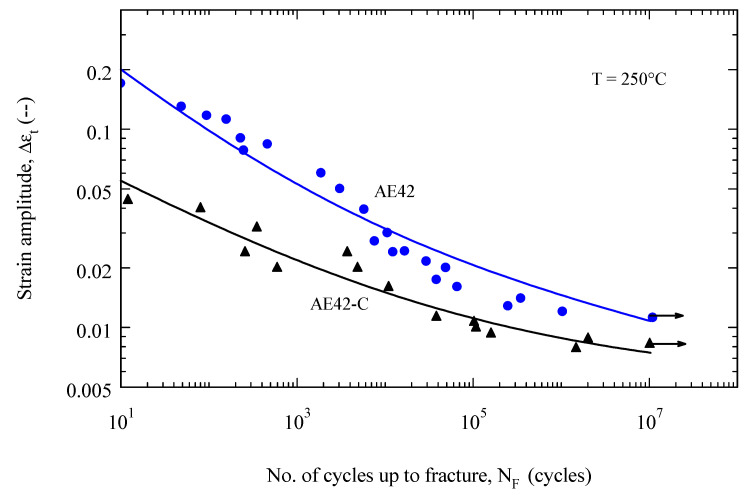
Fatigue life of AE42-C and the unreinforced AE42 at 250 °C.

**Figure 11:**
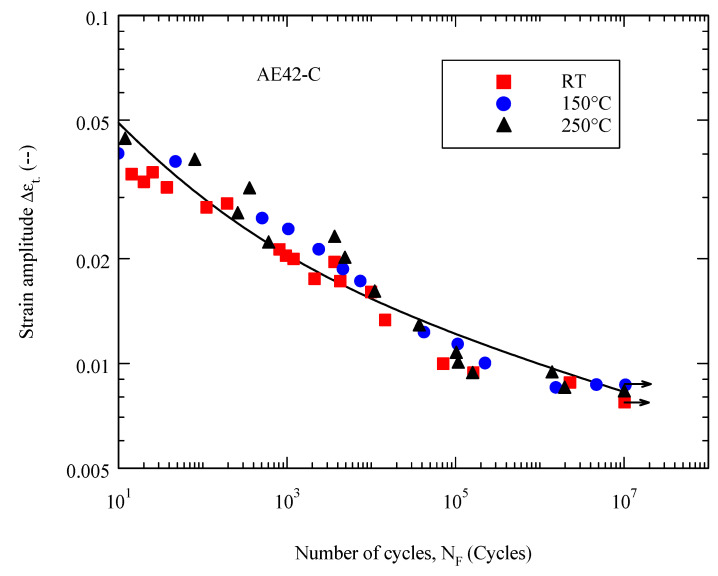
Fatigue life curves of AE42-C at temperatures 20 °C, 150 °C, and 250 °C.

The fatigue life curves Δ*ε_t_ (N_F_)* of the composite materials and those of the corresponding unreinforced AE42 are shown in Figure 10 and Figure 11. The fatigue curves Δ*ε_t_ (N_F_)* were described using Equation (4) with the initial proposed values of the parameters *n* and *m* (0.1 and 0.5, respectively) and the accurate values of these parameters were drawn accordingly. The value of n ranged between 0.1 and 0.12, while m was in the range of 0.32 to 0.38. The determined parameters are in the range of the published values [60].

In the LCF range, it was found that at a certain strain amplitude, the composite materials have a lower service life than the unreinforced AE42 (Figure 10), this can be explained by their reduced ductility. At 250 °C, the fatigue life of the AE42 composite is actually lower when compared to the AE42 alloy. This can be explained by the increased brittleness of the AE42 composite due to the addition of short carbon fibers. This increase in the brittleness of the AE42 composite, as indicated in the fracture surfaces shown in Figure 8 and Figure 9, results in a decrease in its total strain amplitude compared with the unreinforced alloy, as shown in Figure 10.

The relationship between strain amplitude and the number of cycles to failure at room temperature, 150 °C, and 250 °C is illustrated in Figure 11. From the increase in temperature, it can be seen that the strain amplitude of the AE42 composite is comparable. Previous studies mentioned that, as the temperature increases up to 200 °C, the largest portion of the strain amplitude switches to plastic strain, which is the main cause of failure [61,62]. This is because the ductility of the composite increases with increasing temperature, resulting in a decrease in strength. In summary, the fatigue life of the AE42 composite will be relatively the same or may also be slightly improved at elevated temperatures despite the decrease in composite strength.

## 4. Conclusions

Based on the achieved results of the tensile and fatigue tests and the SEM investigation of the unreinforced AE42 and AE42-C, the following main conclusions can be drawn:

1- Reinforcing AE42 using carbon fibers has doubled the tensile yield stress at room temperature and further increased it at higher temperatures, reaching 2.5 times that of the unreinforced AE42 at 300 °C. While the early fracture of the composites has limited the improvement in the ultimate tensile strength.
2- The reinforcement of Mg alloy AE42 with short carbon fibers has markedly improved the fatigue strength at the HCF range (10^7^ cycles) from 25 MPa to 52.7 MPa at 250 °C. The composites showed more increased fatigue strength (68 MPa) at room temperature.
3- The slight temperature influence on the fatigue behavior of composite AE42-C up to 250 °C reflects its thermal stability.
4- Fiber breakage and fiber detachment were features of the fracture surface of the composites under tensile and fatigue load. In addition, fatigue serrations and limited dimples were detected at the metallic magnesium bridges.
5- The fatigue life curves Δ*ε_t_ (N_F_)* of the unreinforced AE42 and the composites AE42-C were well described using the Basquin and Manson–Coffin approaches.

## Figures and Tables

**Table 1:** Chemical composition of the unreinforced AE42.

Elements	Al	Zn	Mn	Ce	La	Nd	Pr	Th	Mg
wt %	3.93	0.01	0.30	1.20	0.60	0.40	0.10	0.26	Bal.

**Table 2:** Fitting parameters of Wohler curves of the unreinforced AE42 and AE42 composite and fatigue strength at $N=10^{7}$ cycles at the testing temperatures.

Material	No. of Experiments	*T* (°C)	*a*	*B*	*c*	Max. Dev.	Standard Error, *r^2^*	Fatigue Strength (MPa) at *N =* 10^7^
Unreinforced AE42	21	250	126	3.34	23.6	10.3	0.986	25
AE42-C	16	250	134	4.24	43.8	3.6	0.997	52.7
	13	150	136	3.7	62.7	4.90	0.996	66.5
	18	20	138	3.7	64.3	7.0	0.990	68

## Data Availability

The data presented in this study are available on request from the corresponding author. The data are not publicly available due to the extremely large size.

## Nomenclature

*N_F_*	Number of cycles to fracture (cycles)
*σ_a_*	Stress amplitude (MPa)
*R*	Fatigue stress ratio
*L_f_*	Fiber length (µm)
*d_f_*	Fiber diameter (µm)
*ν_f_*	Volume fraction
RE	Rare earth elements such as Ce, La, bd, Pr, …
LCF	Low-cycle fatigue
HCF	High-cycle fatigue
*ε*	Strain (%)
σ_0.2_	Yield stress (MPa)
σ_UTS_	Ultimate tensile strength (MPa)
*a*	A fitting parameter
*b*	A fitting parameter
*c*	A fitting parameter
Δ$\varepsilon_{el}$	Elastic strain region (--)
Δ$\varepsilon_{pl}$	Plastic strain region (--)
Δ$\varepsilon_{t}$	Total strain (--)
*E*	Temperature-dependent elastic modulus
ξ	Parameter with a value of 3.5
*A*	A constant
*B*	A constant
*RT*	Room temperature (°C)
*n*	A constant with a value of 0.1
*M*	A constant with a value of 0.5

## References

[1] Kulkarni S., Edwards D.J., Parn E.A., Chapman C., Aigbavboa C.O., Cornish R. (2018). Evaluation of Vehicle Lightweighting to Reduce Greenhouse Gas Emissions with Focus on Magnesium Substitution. J. Eng. Des. Technol..

[2] Czerwinski F. (2021). Current Trends in Automotive Lightweighting Strategies and Materials. Materials.

[3] Liu B., Yang J., Zhang X., Yang Q., Zhang J., Li X. (2023). Development and Application of Magnesium Alloy Parts for Automotive OEMs: A Review. J. Magnes. Alloy..

[4] Thiagarajan C., Lakshminarayanan N., Anand A., Nikhil Santhosh M., Anderson N.J. (2020). Investigation and Analysis of Properties of Magnesium Alloy for Suitability to Electric Vehicle Components. IOP Conf. Ser. Mater. Sci. Eng..

[5] Minárik P., Král R., Čížek J., Chmelík F. (2016). Effect of Different c/a Ratio on the Microstructure and Mechanical Properties in Magnesium Alloys Processed by ECAP. Acta Mater..

[6] Harhash M., Ataya S., Abd El Hady M., El Mahallawy N. (2014). Microstructural Characterization and Kinetics of Diffusion Bonded AZ31/Al by Hot Press Cladding. Materwiss. Werksttech..

[7] Wu X., Zhao C., Liu X. (2019). Effect of Ca Addition on the Microstructure and Tensile Property of AE42 Alloy. Mater. Lett..

[8] Mondal A.K., Blawert C., Kumar S. (2015). Corrosion Behaviour of Creep-Resistant AE42 Magnesium Alloy-Based Hybrid Composites Developed for Powertrain Applications. Mater. Corros..

[9] Zhai W., Bai L., Zhou R., Fan X., Kang G., Liu Y., Zhou K. (2021). Recent Progress on Wear-Resistant Materials: Designs, Properties, and Applications. Adv. Sci..

[10] Mo N., Tan Q., Bermingham M., Huang Y., Dieringa H., Hort N., Zhang M.X. (2018). Current Development of Creep-Resistant Magnesium Cast Alloys: A Review. Mater. Des..

[11] Ashrafizadeh S.M., Mahmudi R. (2019). Effects of Gd, Y, and La Rare-Earth Elements on the Microstructural Stability and Elevated-Temperature Mechanical Properties of AZ81 Magnesium Alloy. Metall. Mater. Trans. A Phys. Metall. Mater. Sci..

[12] Kim J., Kawamura Y. (2013). Influence of Rare Earth Elements on Microstructure and Mechanical Properties of Mg97Zn1Y1RE1 Alloys. Mater. Sci. Eng. A.

[13] Ganguly S., Mondal A.K. (2018). Influence of SiC Nanoparticles Addition on Microstructure and Creep Behavior of Squeeze-Cast AZ91-Ca-Sb Magnesium Alloy. Mater. Sci. Eng. A.

[14] Li C.D., Wang X.J., Liu W.Q., Shi H.L., Ding C., Hu X.S., Zheng M.Y., Wu K. (2014). Effect of Solidification on Microstructures and Mechanical Properties of Carbon Nanotubes Reinforced Magnesium Matrix Composite. Mater. Des..

[15] Ercetin A., Pimenov D.Y. (2021). Microstructure, Mechanical, and Corrosion Behavior of Al2o3 Reinforced Mg2zn Matrix Magnesium Composites. Materials.

[16] Shao J., Li W., Wang R., Tao Y., Kou H., Deng Y., Zhang X., Li Y., Wang X. (2018). Temperature Dependent Compressive Yield Strength Model for Short Fiber Reinforced Magnesium Alloy Matrix Composites. J. Mater. Sci..

[17] Ataya S., El-Sayed Seleman M.M., Latief F.H., Ahmed M.M.Z., Hajlaoui K., Elshaghoul Y.G.Y., Habba M.I.A. (2022). Microstructure and Mechanical Properties of AZ91 Rein-Forced with High Volume Fraction of Oriented Short Carbon Fibers. Materials.

[18] Yang Z., Xu H., Wang Y., Hu M., Ji Z. (2019). Microstructures and Mechanical Properties of SCF/AZ31B Composites Fabricated by Multi-Times Hot-Extrusion. Results Phys..

[19] Ataya S., El-Magd E. (2007). Modeling the Creep Behavior of Mg Alloys with and without Short-Fiber Reinforcement. Comput. Mater. Sci..

[20] Xu H., Yang Z., Hu M., Ji Z. (2020). Effect of Short Carbon Fiber Content on SCFs/AZ31 Composite Microstructure and Mechanical Properties. Results Phys..

[21] Ataya S., Alsaleh N.A., El-Sayed Seleman M.M. (2019). Strength and Wear Behavior of Mg Alloy AE42 Reinforced with Carbon Short Fibers. Acta Metall. Sin. Engl. Lett..

[22] Teschke M., Koch A., Walther F. (2020). Comparison of High-Temperature Compression and Compression-Compression Fatigue Behavior of Magnesium Alloys DieMag422 and AE42. Materials.

[23] Shih T.S., Liu W.S., Chen Y.J. (2002). Fatigue of As-Extruded AZ61A Magnesium Alloy. Mater. Sci. Eng. A.

[24] Ghorbanpour S., McWilliams B.A., Knezevic M. (2020). Effects of Environmental Temperature and Sample Pre-Straining on High Cycle Fatigue Strength of WE43-T5 Magnesium Alloy. Int. J. Fatigue.

[25] Park S.H. (2017). Effect of Initial Twins on the Stress-Controlled Fatigue Behavior of Rolled Magnesium Alloy. Mater. Sci. Eng. A.

[26] Jabbari A.H., Sedighi M., Jahed H., Sommitsch C. (2020). Low Cycle Fatigue Behavior of AZ31B Extrusion at Elevated Temperatures. Int. J. Fatigue.

[27] Wang B.J., Wang S.D., Xu D.K., Han E.H. (2017). Recent Progress in Fatigue Behavior of Mg Alloys in Air and Aqueous Media: A Review. J. Mater. Sci. Technol..

[28] Kim Y.J., Kim S.-H., Lee J.U., You B.S., Park S.H. (2017). Evolution of High-Cycle Fatigue Behavior of Extruded AZ91 Alloy by Artificial Cooling during Extrusion. Mater. Sci. Eng. A.

[29] Li F., Wang Y., Chen L., Liu Z., Zhou J. (2005). Low-Cycle Fatigue Behavior of Two Magnesium Alloys. J. Mater. Sci..

[30] Wittke P., Klein M., Walther F. (2019). Mechanism-Oriented Characterization of the Load Direction-Dependent Cyclic Creep Behavior of the Magnesium Alloys Mg-4Al-2Ba-2Ca and AE42 at Room Temperature. Eng. Fail. Anal..

[31] Dieringa H., Huang Y., Wittke P., Klein M., Walther F., Dikovits M., Poletti C. (2013). Compression-Creep Response of Magnesium Alloy DieMag422 Containing Barium Compared with the Commercial Creep-Resistant Alloys AE42 and MRI230D. Mater. Sci. Eng. A.

[32] Klein M., Buhr P., Walther F. (2016). Microstructure-Based Assessment of the Corrosion Fatigue Behavior of the Creep-Resistant DieMag422 and AE42 Magnesium Alloys. Solid State Phenom..

[33] Macek W., Tomczyk A., Branco R., Dobrzyński M., Seweryn A. (2023). Fractographical Quantitative Analysis of EN-AW 2024 Aluminum Alloy after Creep Pre-Strain and LCF Loading. Eng. Fract. Mech..

[34] Ahmed M.M.Z., Alzahrani B., Jouini N., Hessien M.M., Ataya S. (2021). The Role of Orientation and Temperature on the Mechanical Properties of a 20 Years Old Wind Turbine Blade GFR Composite. Polymers.

[35] ASTM International (2010). ASTM E8 ASTM E8/E8M Standard Test Methods for Tension Testing of Metallic Materials.

[36] ASTM International (2002). ASTM Standard Practice for Conducting Force Controlled Constant Amplitude Axial Fatigue Tests of Metallic Materials.

[37] Choi J., Lee J., Jun N., Seok C.-S., Park S., Kim G. (2019). Development of Laboratory Fatigue Testing Apparatus for Automotive Vehicle Engine Valve Simulating Actual Operating Conditions. Int. J. Precis. Eng. Manuf..

[38] Ataya S., El-Sayed Seleman M.M., Latief F.H., Ahmed M.M.Z., Hajlaoui K., Soliman A.M., Alsaleh N.A., Habba M.I.A. (2022). Wear Characteristics of Mg Alloy AZ91 Reinforced with Oriented Short Carbon Fibers. Materials.

[39] Xue S., Sun Y.S., Ding S.S., Bai Q., Bai J. (2005). Effects of Calcium Additions on Microstructure and Creep Behaviour of AE42 Alloy. Mater. Sci. Technol..

[40] Wang M., Jin P., Wang J. (2014). Hot Deformation and Processing Maps of 7005 Aluminum Alloy. High Temp. Mater. Process..

[41] Wang J., Shi B., Yang Y. (2014). Hot Compression Behavior and Processing Map of Cast Mg-4Al-2Sn-Y-Nd Alloy. Trans. Nonferrous Met. Soc. China.

[42] Wang M., Jin P., Wang J., Han L., Cui C. (2014). Hot Deformation Behavior and Workability of (SiCp + Mg2B2O5w)/6061 Al Hybrid and SiCp/6061 Al Composites. Acta Metall. Sin. Engl. Lett..

[43] Li S., Qi L., Zhang T., Zhou J., Li H. (2015). Interfacial Microstructure and Tensile Properties of Carbon Fiber Reinforced Mg-Al-RE Matrix Composites. J. Alloys Compd..

[44] Nai M.H., Wei J., Gupta M. (2014). Interface Tailoring to Enhance Mechanical Properties of Carbon Nanotube Reinforced Magnesium Composites. Mater. Des..

[45] Viala J.C., Fortier P., Claveyrolas G., Vincent H., Bouix J. (1991). Effect of Magnesium on the Composition, Microstructure and Mechanical Properties of Carbon Fibres. J. Mater. Sci..

[46] Doiphode R.L., Murty S.V.S.N., Prabhu N., Kashyap B.P. (2015). Grain Growth in Calibre Rolled Mg–3Al–1Zn Alloy and Its Effect on Hardness. J. Magnes. Alloy..

[47] Feldhoff A., Pippel E., Woltersdorf J. (1999). Carbon-Fibre Reinforced Magnesium Alloys: Nanostructure and Chemistry of Interlayers and Their Effect on Mechanical Properties. J. Microsc..

[48] Jayalakshmi S., Kailas S., Seshan S., Kim K.B., Fleury E. (2006). Tensile Strength and Fracture Toughness of Two Magnesium Metal Matrix Composites. J. Ceram. Process. Res..

[49] Ahmed M.M.Z., El-Sayed Seleman M.M., Albaijan I., Abd El-Aty A. (2023). Microstructure, Texture, and Mechanical Properties of Friction Stir Spot-Welded AA5052-H32: Influence of Tool Rotation Rate. Materials.

[50] Pettersen G., Westengen H., Høier R., Lohne O. (1996). Microstructure of a Pressure Die Cast Magnesium—4 wt.% Aluminium Alloy Modified with Rare Earth Additions. Mater. Sci. Eng. A.

[51] Srivatsan T.S., Al-Hajri M., Lam P.C. (2005). The Quasi-Static, Cyclic Fatigue and Final Fracture Behavior of a Magnesium Alloy Metal-Matrix Composite. Compos. Part B Eng..

[52] Murashkin M., Sabirov I., Prosvirnin D., Ovid’ko I., Terentiev V., Valiev R., Dobatkin S. (2015). Fatigue Behavior of an Ultrafine-Grained Al-Mg-Si Alloy Processed by High-Pressure Torsion. Metals.

[53] Forsyth P.J.E. (1963). Fatigue Damage and Crack Growth in Aluminium Alloys. Acta Metall..

[54] Basquin O.H. (1910). The Experimental Law of Endurance Test. Am. Soc. Test. Mater. Proc..

[55] Coffin L.F. (1954). A study of the effects of cyclic thermal stresses on a ductile metal. Trans. ASME.

[56] Manson S.S. (1966). Interfaces between Fatigue, Creep, and Fracture. Int. J. Fract. Mech..

[57] Manson S.S. (1965). Fatigue: A Complex Subject—Some Simple Approximations. Exp. Mech..

[58] Hamada A.S., Järvenpää A., Ahmed M.M.Z., Jaskari M., Wynne B.P., Porter D.A., Karjalainen L.P. (2015). The Microstructural Evolution of Friction Stir Welded AA6082-T6 Aluminum Alloy during Cyclic Deformation. Mater. Sci. Eng. A.

[59] Zenner H. (1976). Niedrig-Lastwechsel-Ermüdung (Low Cycle Fatigue).

[60] Buxbaum O. (1992). Betriebsfestigkeit. Sichere und Wirtschaftliche Bemessung Schwingbruchgefährdeter Bauteile. 2.Erw.Aufl..

[61] Wang C.Y., Liu Z., Chen L.J. (2007). Low-Cycle Fatigue Behavior of Three Die Cast Magnesium Alloys. Mater. Sci. Forum.

[62] Huppmann M., Lentz M., Brömmelhoff K., Reimers W. (2010). Fatigue Properties of the Hot Extruded Magnesium Alloy AZ31. Mater. Sci. Eng. A.

